# Reusable Plastic Crates (RPCs) for Fresh Produce (Case Study on Cauliflowers): Sustainable Packaging but Potential *Salmonella* Survival and Risk of Cross-Contamination

**DOI:** 10.3390/foods10061254

**Published:** 2021-06-01

**Authors:** Francisco López-Gálvez, Laura Rasines, Encarnación Conesa, Perla A. Gómez, Francisco Artés-Hernández, Encarna Aguayo

**Affiliations:** 1 Postharvest and Refrigeration Group, Escuela Técnica Superior de Ingeniería Agronómica (ETSIA), Universidad Politécnica de Cartagena (UPCT), Paseo Alfonso XIII, 48, 30203 Cartagena, Spain; francisco.lopezgalvez@upct.es (F.L.-G.); laura.rasines@upct.es (L.R.); fr.artes-hdez@upct.es (F.A.-H.); 2 Food Quality and Health Group, Institute of Plant Biotechnology (UPCT), Campus Muralla del Mar, 30202 Cartagena, Spain; perla.gomez@upct.es; 3 Plant Production Department, ETSIA, Institute of Plant Biotechnology (UPCT), Paseo Alfonso XIII, 48, 30203 Cartagena, Spain; encarnacion.conesa@upct.es

**Keywords:** pathogenic bacteria, food contact surface, transfer, *Brassica*, life cycle analysis, wooden boxes, environmental impact

## Abstract

The handling of fresh fruits and vegetables in reusable plastic crates (RPCs) has the potential to increase the sustainability of packaging in the fresh produce supply chain. However, the utilization of multiple-use containers can have consequences related to the microbial safety of this type of food. The present study assessed the potential cross-contamination of fresh cauliflowers with *Salmonella enterica* via different contact materials (polypropylene from RPCs, corrugated cardboard, and medium-density fiberboard (MDF) from wooden boxes). Additionally, the survival of the pathogenic microorganism was studied in cauliflowers and the contact materials during storage. The life cycle assessment (LCA) approach was used to evaluate the environmental impact of produce handling containers made from the different food-contact materials tested. The results show a higher risk of cross-contamination via polypropylene compared with cardboard and MDF. Another outcome of the study is the potential of *Salmonella* for surviving both in cross-contaminated produce and in contact materials under supply chain conditions. Regarding environmental sustainability, RPCs have a lower environmental impact than single-use containers (cardboard and wooden boxes). To exploit the potential environmental benefits of RPCs while ensuring food safety, it is necessary to guarantee the hygiene of this type of container.

## 1. Introduction

Reusable plastic crates (RPCs) are utilized in different steps of the fruit and vegetable supply chain, including harvest, handling, packaging, and transport operations, as well as in the retail sector [1]. The use of RPCs for the handling of fresh produce has some advantages, such as the potential to improve environmental sustainability [2]. On the other hand, different studies have raised awareness regarding the hygienic status of RPCs and their possible role as a source of microbiological contamination [3,4,5].

Fruits and vegetables are increasingly being recognized as a source of foodborne outbreaks [6,7]. Pathogenic microorganisms can survive in fresh produce throughout the supply chain, thereby posing a risk to consumers [8]. Cauliflower-containing products have faced recalls due to the potential presence of pathogenic bacteria [9]. Zhang et al. [10] detected *L. monocytogenes* in fresh-cut cauliflower (florets). Quiroz-Santiago et al. [11] detected *Salmonella* in 9% of the cauliflower samples they analyzed (*n* = 100). The contamination of fresh produce can come from several sources, including food-contact surfaces where pathogenic microorganisms can survive and be transferred to food [5]. Bacterial transfer between contact surfaces and food and vice versa is influenced by many factors, including the bacterial species, handling of the inoculum, degree of contamination, type of surface, type of food, temperature, moisture, duration of the contact, and pressure [12,13]. The transfer of microorganisms between fresh produce and equipment surfaces (including harvest bins and packaging boxes or crates) is significant [14,15]. Although the use of RPCs has not been linked directly with foodborne outbreaks, indirect evidence indicates that there is a potential risk when hygiene fails to be properly maintained [1]. Inadequate cleaning can enhance *Salmonella* survival in plastic containers used in harvest operations [16]. The presence of fresh produce residues (e.g., intact tissues, organic matter, decaying plant material) can enable growth and biofilm formation by *Salmonella* in food-contact surfaces [17]. Furthermore, different studies have suggested that there is a higher transfer of microorganisms to fresh produce from plastic containers in comparison with containers made of other materials. Patrignani et al. [18] showed a higher transfer of bacteria from RPCs to peaches compared with cardboard, hypothesizing that such a difference would be caused by the higher entrapment capability of cardboard. Aviat et al. [19] observed a higher transfer of *E. coli* to apples from polypropylene surfaces compared with wood and cardboard surfaces. In their study, apart from the higher entrapping capability of wood and cardboard compared with plastic, the authors also suggested the ability of microorganisms to form biofilms on plastic surfaces as a potential cause for the differences with the other materials. The study by Siroli et al. [20] also indicated that the risk of microbial cross-contamination is higher via plastic surfaces than via cardboard surfaces.

In the present study, events of cross-contamination between inoculated (*Salmonella enterica*) and non-contaminated cauliflowers via different contact materials were simulated and assessed. The materials tested were: polypropylene from RPCs, corrugated cardboard, and medium-density fiberboard (MDF) from wooden boxes. These materials are commonly used in the manufacturing of fresh-produce handling containers. The survival of the pathogenic microorganism in the vegetable and on the contact surfaces under supply chain conditions was also evaluated. Furthermore, a life cycle assessment (LCA) approach was used to evaluate the sustainability of packaging containers made of the different materials studied.

## 2. Materials and Methods

### 2.1. Transfer and Survival of Salmonella via Different Fresh-Produce Container Materials

#### 2.1.1. Via Polypropylene

##### Salmonella Strains and Inoculum Preparation

Three *Salmonella enterica* subsp. *enterica* strains (CECT 443, CECT 4141, and CECT 4372) were used for the preparation of the inoculum. Starting from a refrigerated stock culture, the strains were grown separately in Tryptic Soy Broth (TSB) for 20 h at 37 °C. Subsequently, a cocktail was prepared by mixing 15 mL of each strain, for a total volume of 45 mL. The cocktail was centrifuged at 4500× *g* for 20 min, the supernatant was discarded, and the cells were resuspended in saline solution (0.85% NaCl). Finally, the *Salmonella* suspension was used to inoculate 5 L of saline solution at room temperature to reach a level of *Salmonella* of ≈10^7^ cfu/mL. The inoculum was used immediately after its preparation.

##### Plant Material and Inoculation

Cauliflowers (*Brassica oleracea var. botrytis* cv. ‘Altair’) provided by Jimbofresh International S.L. (La Unión, Murcia, Spain) were used in the experiment. These mini-cauliflowers are harvested when the diameter of the head is in the range of 8–11 cm, so they are smaller than regular cauliflowers (harvested when they reach a diameter of 15–25 cm) [21]. Detailed information on the dimensions of the cauliflowers used in the experiment can be found in the Supplementary Materials (Table S1). After harvesting (the day before the experiment), the cauliflowers were stored under refrigeration (4 °C). On the day of the experiment, they were taken out of the cold room and allowed to reach room temperature before inoculation. The curds were then immersed (only the apical half) in the inoculated saline solution for 1 min. After draining thoroughly, they were dried for 2 h in a biosafety cabinet until no visible liquid remained on or between the florets. By measuring the weight difference, it was estimated that a mean volume of 4.5 mL of the *Salmonella* suspension was withheld by each cauliflower head after inoculation and draining, and before drying.

##### Cross-Contamination

**Figure 1:**
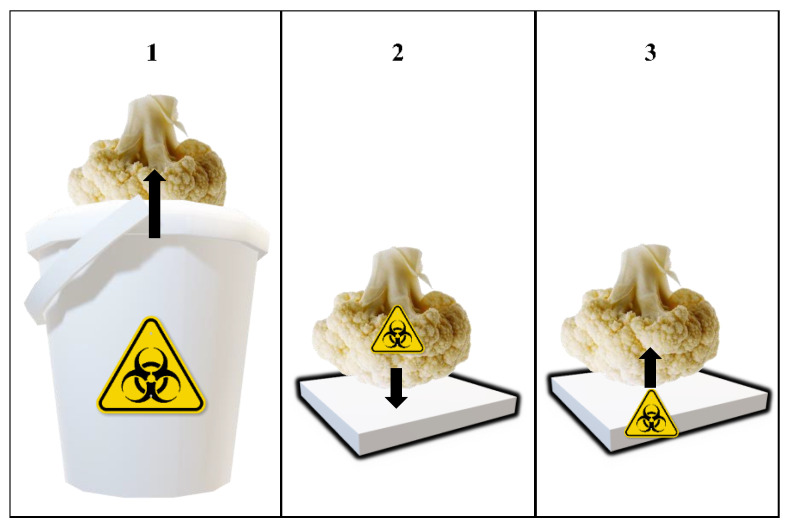
Schematic depiction of the cross-contamination events simulated in the lab. Step 1: Dip inoculation of cauliflowers; Step 2: Transfer of *Salmonella* from inoculated cauliflowers to container pieces; Step 3: Transfer of *Salmonella* from container pieces to non-inoculated cauliflowers. Black arrows represent the direction of the transfer of *Salmonella* cells.

Figure 1 shows the steps of the simulated cross-contamination events. The square polypropylene (PP) pieces (3.5 × 3.5 = 12.25 cm^2^) utilized in the experiment were obtained by cutting RPCs used for the handling of fruits and vegetables. They were washed using water and dishwasher, rinsed with distilled water, and sterilized by autoclaving before the experiments. The inoculated cauliflowers were placed on top of the sterile PP fragments for 1 h at room temperature to permit the transfer of the inoculated bacteria. The same contact time has been used in other studies assessing microbial cross-contamination of fresh produce via handling container surfaces [19,22]. The cauliflowers were placed upside down, to allow for contact of the inoculated area (apical half) with the PP. Afterwards, the inoculated cauliflowers were removed, and non-inoculated cauliflowers were immediately placed on top of the PP pieces, in the same position (the apical part in contact with the PP). Once again, a contact time of 1 h was used to allow for the transfer of bacteria from the PP surface to the cauliflowers. The cross-contamination of these cauliflowers was studied. Temperature and relative humidity (RH) during these cross-contamination steps were monitored using a thermometer and a psychrometer, respectively. A test was performed to evaluate the real contact area between cauliflowers and the PP pieces [13]. In this test, the apical part of cauliflowers (*n* = 24) was placed in contact with a permanent black ink pad, and then immediately placed on top of PP pieces. Photographs of the stained pieces were taken using a camera, and the blackened area of the PP pieces was measured using the image processing software ImageJ (National Institutes of Health, Bethesda, MD, USA) [23].

##### Storage

Inoculated and cross-contaminated cauliflowers were packaged separately in polyethylene terephthalate (PET) trays covered with perforated polyethylene (PE) film as usually performed by the fresh produce industry. Packaged cauliflowers were stored in a cold room at 4 °C for seven days to simulate the storage and transport conditions, followed by six days at 8 °C to simulate supermarket and household conditions. The PP pieces were placed on trays with the inoculated side facing upwards and were stored in the same cold room used for the cauliflowers.

##### Sampling and Microbiological Analysis

Table 1 shows the types of samples that were analyzed at different moments during the experiment. At each sampling time, three independent samples from each sample type were analyzed. In the case of the cauliflowers, the apical half was cut, and 50 g was taken aseptically for analysis. After adding 200 mL of buffered peptone water (BPW, 20 g/L) to the sample (dilution 1 in 5), it was homogenized using a stomacher for 1 min. The presence of *Salmonella* in the PP pieces and PE films was analyzed using sterile cotton swabs (Aptaca Spa, Canelli, Italy) wetted in BPW (20 g/L). In the case of the PP pieces, the whole area (12.25 cm^2^) was swabbed. In the case of the PE films, an area of ≈100 cm^2^ (10 × 10 cm) of the zone in contact with the apical area of the inoculated or contaminated cauliflower was swabbed. Swabbing was performed in a standardized way regarding the number and the direction of swab passes. In both cases, swabs were placed in test tubes containing 9 mL of BPW (20 g/L) after use. Serial dilutions in BPW (2 g/L) were prepared as needed, and samples were plated in Xylose Lysine Deoxycholate Agar (XLD; Scharlab, Barcelona, Spain). Apart from the direct plating, an enrichment of the samples was performed by incubation at 37 °C for 24 h. After incubation, the enrichment was also plated in XLD, and the plates were incubated at 37 °C for 24 h before interpretation of results. Red colonies with a black center were considered to be *Salmonella* spp. The detection limit before enrichment was 5 cfu/g in cauliflower, 0.7 cfu/cm^2^ in the PP pieces, and 0.09 cfu/cm^2^ in the packaging film.

#### 2.1.2. Effect of the Inoculum Size

The impact of lower inoculum sizes on the transfer from the inoculated product to the PP surface and on the subsequent cross-contamination of uncontaminated cauliflower was studied. The setup of the experiment was similar to that described in Section 2.1.1 albeit with some modifications. In this case, no storage was performed as the goal was to assess if lower inoculum levels could also lead to cross-contamination. In contrast with the inoculated saline solution prepared in the previous experiments (≈10^7^ cfu/mL), in this case, two saline solutions containing a level of ≈10^6^ cfu/mL and ≈10^4^ cfu/mL of *Salmonella* were prepared for the inoculation of the cauliflowers. Five independent samples from each sample type were analyzed at each sampling time.

#### 2.1.3. Via Cardboard and MDF

Transfer and survival of the pathogenic microorganism via other materials were assessed. Cardboard and fiberboard (medium-density fiberboard (MDF)) from wooden boxes were tested as materials commonly used in the manufacturing of vegetable handling containers [24]. The experimental setup was as described in Section 2.1.1 with modifications. In this case, the pieces could not be washed or sterilized by autoclaving but were sanitized by exposure (both sides) to UV light in a biosafety cabinet for 1 h as in Li et al. [25]. In this experiment, the survival of *Salmonella* during storage was assessed in cross-contaminated cauliflower and the container pieces, but not in the inoculated cauliflower or the PE films. In this test, the analysis of cauliflower and pieces during storage was performed at three time points (after 1, 6, and 13 days of storage).

#### 2.1.4. Statistical Analysis

The statistical analyses were executed using IBM SPSS statistics version 26. A level of statistical significance of *p* < 0.05 was used. Data on microbial populations were log-transformed. The Shapiro-Wilk test and Levene’s test were used to assess the normality and the homogeneity of variance, respectively. When normality could be assumed, *t*-tests or One-way ANOVA were used to compare treatments, using Tukey’s HSD or Dunnett’s as post hoc tests depending on the homogeneity of the variances. For data not following a normal distribution, non-parametric tests (Mann–Whitney U and Kruskal–Wallis) were used to search for differences between treatments. Binary logistic regression was used for the analysis of presence/absence data.

### 2.2. Environmental Impact of Different Types of Fresh Produce Handling Containers

A life cycle assessment (LCA) was performed according to the ISO standards 14040 and 14044 [26,27] using Product Category Rules for Crates for Food [28]. LCA includes four stages: (1) Goal and scope definition, (2) Inventory analysis, (3) Impact assessment, and (4) Interpretation. In LCA studies, the functional unit (FU) is used to normalize all the inputs and outputs. The functional unit in this study was defined as the distribution of 1 kg of cauliflowers in plastic crates, wooden boxes, or cardboard boxes.

#### 2.2.1. Goal and Scope

**Figure 2:**
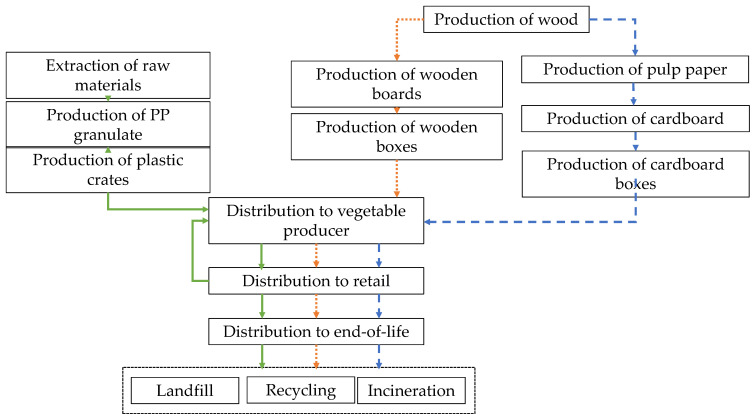
System boundaries of reusable plastic crates (RPCs, polypropylene, green arrows) and single-use cardboard (blue dashed arrows) and wooden (orange dotted arrows) boxes.

The goal of this LCA was to compare the environmental impact of reusable plastic crates (RPCs, polypropylene) with that of single-use cardboard (corrugated cardboard) and wooden boxes (poplar wood + pinewood + MDF) using the LCA methodology. Regarding the system boundaries, upstream, core, and downstream processes must be defined. Figure 2 shows the system boundaries of the different types of boxes. In relation to the upstream processes, for wooden and cardboard boxes the life cycle starts in forestry agriculture (production of plants and extraction of resources), while for plastic crates it starts in the extraction of resources and the production of polymer. The next step, in all cases, is the transport of the raw materials to the core process. The core stage covers the manufacture of the final product, including the use of fuel and electricity, emissions generated during manufacturing, machinery maintenance, and treatment of the residues. The final stage (downstream) includes the transport to final disposal and waste treatment, which is different for each type of box.

#### 2.2.2. Life Cycle Inventory

Cradle-to-grave LCA was performed considering a total of 150 rotations (uses) for plastic crates ([2], and personal communication from a RPC managing company). In other words, it was assumed that each plastic crate is cleaned and reused 150 times, while wood and cardboard boxes are not reused, and it is necessary to produce new boxes for subsequent shipments. Spanish law establishes that wooden and cardboard packaging used for fresh food, regardless of whether they are primary or secondary packaging, can only be used once [29]. In all the cases, the dimensions of the boxes were 40 × 60 × 12 (width × length × height) in cm, and the inner volume was 28.8 L. Each box, regardless of the building material, can be used to carry 6 kg of cauliflower. The list of materials consumed for the manufacturing of each type of box is listed in Table 2. The plastic crates assessed in this LCA are made using primary granulated polypropylene only. The materials in the cardboard boxes evaluated are recycled cardboard (35% in weight) and virgin cardboard (65%). Finally, for the manufacture of the wooden boxes assessed, medium-density fiberboard (MDF) (65.1%), pinewood (20.8%), poplar wood (13.9%), and stainless steel (0.2%) are used. The different transport steps assumed are shown in Table 3. These transport steps included: the shipment of materials to the box manufacturing centers; the transport of crates/boxes to fresh produce packing-houses and retail centers; the return of plastic crates to the cleaning centers; and the transport of crates/boxes to the end-of-life steps. In the case of RPCs, apart from the material for crate production, and the transport steps, the energy and water consumption requirements for the cleaning of the crates during the 150 rotations before disposal were also considered. Based on technical data sheets from RPC washing tunnels [30,31], it was assumed that for the washing of one plastic crate, 0.4 L of water, 0.2% of caustic detergent, and 0.04 kWh of energy are needed. Moreover, scenarios for the waste disposal of the different types of boxes were assessed according to the Spanish annual report on the generation and management of waste [32]. For plastic crates, it was assumed that 79% are recycled, 17% go to landfill, and 4% are incinerated. A total of 65% of cardboard boxes are recycled and 35% go to incineration. Finally, in the case of wooden boxes, 87% of pine and poplar tables are recycled to obtain particle board, 3% finish in the landfill, and 10% are incinerated, whilst in the case of MDF, 77% is incinerated and 23% ends in the landfill.

#### 2.2.3. Impact Assessment

The LCA was performed using SimaPro 9.1 software (PRé Sustainability, Amersfoort, The Netherlands) [36] with the Ecoinvent 3.6 database (Ecoinvent, Zurich, Switzerland) [37]. The CML baseline (Institute of Environmental Sciences, Leiden University, The Netherlands) (Global warming potential, ozone layer depletion, photochemical oxidation, abiotic depletion, acidification eutrophication, freshwater, marine aquatic, and terrestrial ecotoxicity, and human toxicity) and Cumulative Energy Demand (CED) methods were applied.

## 3. Results

### 3.1. Transfer and Survival of Salmonella in Cauliflowers via Different Container Materials

#### 3.1.1. Via Polypropylene

##### Transfer

The level of *Salmonella* detected in the inoculated cauliflowers before contact with the PP fragments was 5.55 ± 0.14 log cfu/g. There were no significant differences between the inoculated cauliflower samples analyzed before and after contact with the PP pieces (*p* > 0.05). This lack of difference is logical, as only a small fraction of the inoculated surface area of each cauliflower was in contact with the PP pieces. Although the total surface of the PP fragments was 12.25 cm^2^, the actual contact area between the PP and the cauliflowers was much smaller due to this vegetable’s uneven surface. The tests performed to elucidate the actual contact surface between cauliflowers and PP pieces showed that the mean global surface contact was 0.5 ± 0.3 cm^2^. Most of the PP pieces analyzed both on the day of the experiment and also during storage showed the presence of *Salmonella* (Table 4). Therefore, transfer of the inoculated microorganism between the inoculated cauliflowers and the PP pieces was detected in most cases. The fact that the pathogenic microorganism was not detected in some of the PP samples could have been due to the limitations of the methods used for the microbiological examination of food contact surfaces [38]. Keeratipibul et al. [39], for example, reported a *Salmonella* recovery efficiency of ≈40% using cotton swabs on dry polyester urethane surfaces. The population of *Salmonella* detected in the PP fragments right after contact with inoculated cauliflower was 0.49 ± 0.71 log cfu/cm^2^ (Table 5).

No *Salmonella* was detected in non-inoculated cauliflowers before contact with the PP pieces (absence after enrichment of samples). After the cross-contamination via the contaminated PP pieces to non-inoculated cauliflowers, in the samples analyzed on the day of the experiment, *Salmonella* was detected only after enrichment (<0.7 log cfu/g). As the pathogenic microorganism was also detected in most cross-contaminated cauliflower samples analyzed during storage (Table 4), we can conclude that there was widespread transfer between the inoculated and non-inoculated cauliflowers via the PP pieces. A larger bacterial transfer from food contact surfaces (plastic, glass, ceramic, stainless steel) to fresh produce than from fresh produce to food contact surfaces had been observed previously [13,40]. More detailed information on the calculations on the transfer of cfu from contact surfaces to non-inoculated cauliflower can be found in the Supplementary Materials (Table S2).

##### Salmonella Survival 

**Figure 3:**
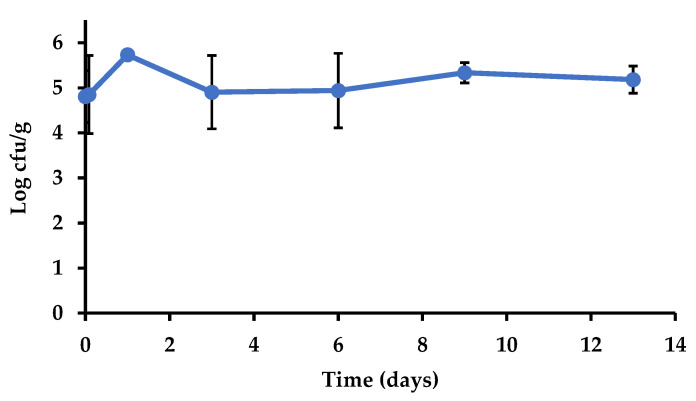
*Salmonella enterica* population (log cfu/g) in inoculated cauliflower during general storage (7 d at 4 °C plus 6 d at 8 °C) in the experiment performed to assess transfer and survival via polypropylene.

The relative humidity measured in the cold room during storage ranged from 73% to 81% at 4 °C, and from 70% to 77% at 8 °C. Figure 3 shows the slight changes in the populations of *Salmonella* in the inoculated cauliflower during refrigerated storage. The levels remained stable without significant changes throughout that period (*p* > 0.05). Additionally, the change in storage temperature from 4 to 8 °C did not lead to changes in *Salmonella* levels in the inoculated cauliflowers. The stability of the populations of *Salmonella* on vegetables stored in the range of temperatures used in this study (4–8 °C) has been observed in other studies. Kroupitski et al. [41] observed minor changes (<0.5 log cfu/g) in *Salmonella* populations after storage of lettuce leaves at 4 °C for nine days, while the results from Delbeke et al. [42] show stability of *Salmonella* populations in basil leaves stored at 7 °C for one week. Pinton et al. [43] observed survival and even growth of the psychrotrophic pathogen *Listeria monocytogenes* on cauliflower and broccoli stored at 4 °C.

Regarding the PP pieces, most of the samples analyzed during the storage were positive for *Salmonella* (Table 4). Throughout the storage, only a few of the positive samples were detected by direct plating (three out of 15 positive samples), whilst the other 12 samples were found positive only after enrichment. Li et al. [24] reported better survival of *Salmonella* in plastic (polyethylene) containers at a refrigeration temperature (3.2 °C; similar to the temperatures used in our tests (4–8 °C)) compared with 22.5 °C.

In the case of the cross-contaminated cauliflowers after contact with contaminated PP, the pathogenic microorganism could not be detected by direct plating during storage, but most of the samples were positive for *Salmonella* after enrichment (Table 4). The proportion of positive samples did not change significantly during storage (*p* > 0.05). These results indicate that, as well as the larger populations present in the inoculated cauliflower (≈5 log cfu/g), the smaller populations present in the cross-contaminated cauliflower (<1 log cfu/g) were able to survive throughout the storage period. Ma et al. [44] reported no effect of the inoculum size (range 0.1–3 log cfu/g) on the survival of *Salmonella* on fresh-cut tropical fruits stored at 4 °C. Strawn and Danyluk [45] also observed stable populations of *Salmonella* on fresh-cut mango inoculated at different initial levels (1, 3, and 5 log cfu/g) and stored at 4 °C.

The packaging film was also analyzed to assess the transfer of *Salmonella* from the inoculated and cross-contaminated cauliflowers to the polyethylene film. In the packaging film from inoculated cauliflowers, *Salmonella* could be detected by direct plating in most of the samples, and only one sample out of 18 was negative both by direct plating and after enrichment. In contrast, in the packaging film from cross-contaminated cauliflowers, no *Salmonella* could be detected by direct plating, and only one sample out of 18 was positive after enrichment (Table 4).

#### 3.1.2. Effect of the Salmonella Inoculum Size

**Figure 4:**
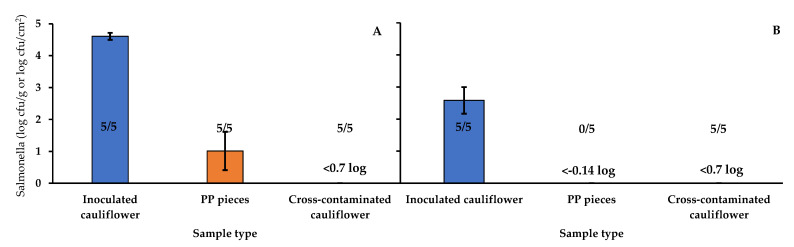
*Salmonella enterica* population (log cfu/g or log cfu/cm^2^) and proportion of positive samples (Number of positive samples/Number of samples analyzed) in different types of samples in the experiment performed to assess the effect of inoculum size on the transfer of the pathogen. (**A**): High inoculum; (**B**): Low inoculum. PP: Polypropylene.

The inoculum size can affect both the number of microorganisms transferred and the transfer rates in the contact between surfaces [46]. Figure 4 shows the results of the test performed to assess the effect of inoculum size on the transfer of *Salmonella* via the PP. The level of *Salmonella* detected in the inoculated cauliflower after drying the inoculum was 4.61 ± 0.11 and 2.58 ± 0.41 log cfu/g for the high and the low inoculum tests, respectively. The PP pieces, after contact with inoculated cauliflower with high inoculum, showed a level of 1.01 ± 0.60 log cfu/cm^2^. In the case of the pieces after contact with low-inoculum cauliflower, the pathogen could not be detected by direct plating or by enrichment. In the case of the cross-contaminated cauliflower, both from high and low inoculum tests, *Salmonella* was not detected by direct plating (<0.7 log cfu/g), but it was detected in all the samples after enrichment. The fact that, in the low inoculum test, the pathogen could be detected in cross-contaminated cauliflower but not in the PP pieces suggests a higher efficiency of recovery of the *Salmonella* cells from cross-contaminated cauliflower compared with the PP pieces.

#### 3.1.3. Via Cardboard and MDF

The level of *Salmonella* detected in the inoculated cauliflowers before contact with the cardboard and MDF pieces was 5.21 ± 0.06 log cfu/g. Regarding the transfer to the different materials, right after contact with the inoculated cauliflowers the cardboard and the MDF pieces showed a mean level of *Salmonella* of 1.14 ± 0.83 and 0.10 ± 0.40 log cfu/cm^2^, respectively (Table 5). In the case of the cross-contaminated cauliflowers analyzed after contact with the cardboard and MDF, no *Salmonella* could be detected by direct plating (<0.7 log cfu/g), but it was detected after enrichment in two out of the three samples, both for cauliflowers cross-contaminated via cardboard and via MDF. The RH in the cold room during storage was between 73% and 81% at 4 °C and between 70% and 77% at 8 °C. In the case of the cardboard pieces, *Salmonella* could be detected by direct plating after one day of storage, but it was detected only after enrichment in the analyses performed after six and 13 days of storage (Table 5). In MDF pieces, the pathogenic microorganism could not be detected by direct plating during storage, but all the samples were positive after enrichment, even after 13 days in the cold room. Regarding the cross-contaminated cauliflowers, *Salmonella* was not detected during storage for both materials; all the samples were negative (by direct plating and also after enrichment) even after only one day of storage (Table 5). The results for the different materials suggest that the transfer of *Salmonella* from the inoculated to non-inoculated cauliflowers was stronger via the PP.

### 3.2. Environmental Impact of Different Types of Fresh Produce Handling Containers

Table 6, Table 7 and Table 8 show the results of the different types of boxes in the various impact categories assessed. The wooden boxes showed a higher environmental impact in all the categories assessed. In the global warming category, we obtained values (per FU) of 0.186, 0.059, and 0.006 kgCO_2_eq for wooden boxes, cardboard boxes, and RPCs, respectively. In all cases, the production step was the stage causing the most greenhouse gas emissions. The materials contributing most to this impact category were the MDF boards used in wooden boxes production, fluting medium and linerboard in cardboard boxes, and obtaining the polypropylene granulate in RPCs. Other activities with a significant contribution to global warming were the end-of-life stage in the case of wooden boxes, and the washing step in the case of the RPCs (electricity and the production of detergent). The contribution of transport in this impact category was negligible in the three cases. The impact of the end-of-life stage of corrugated boxes in the global warming category was negative (this stage reduces the emissions) due to the cardboard production avoided by the recycling process.

The ozone layer depletion and photochemical ozone oxidation had the same behavior as global warming in cardboard and wooden boxes, i.e., the step that showed the largest impact was production, followed by the end-of-life. In the end-of-life scenario of wooden boxes, the MDF boards contributed to a higher extent to ozone layer depletion while the pine and poplar boards contributed to a higher extent to photochemical ozone oxidation. In RPCs, the major contribution in ozone layer depletion was caused by the detergent used for the cleaning of the crates, while the photochemical oxidation category was affected mainly by the RPC production and cleaning process.

The box production step was the major contributor to the acidification and eutrophication potential categories, except in the case of plastic boxes, in which the cleaning process had a larger impact on eutrophication than the RPC production.

Regarding abiotic depletion, the major impacts were caused by MDF in the case of the wooden boxes and by corrugated cardboard production in the cardboard boxes. In both cases, the contribution of these materials exceeded 90%. In the case of RPCs, the production step was the main contributor in the abiotic depletion category, followed by the cleaning step (mainly because of the Spanish electric mix used in this phase).

Environmental ecotoxicity is divided into freshwater, marine, and terrestrial ecotoxicity. The production of boxes and crates affected the marine ecotoxicity category, whilst the washing of RPCs affected the fresh water and terrestrial ecotoxicity. Independently of the type of packaging, the transport had a greater impact on marine ecotoxicity. In contrast to wooden boxes, the end-of-life step of RPCs and cardboard boxes led to a reduction in the environmental ecotoxicity impact. The end-of-life scenario of wooden boxes mainly affected the marine ecotoxicity due to the incineration of the MDF boards.

During the life cycle of boxes and crates, there are emissions of chemical compounds that are toxic to human beings (human toxicity category). In the case of wooden boxes, the toxic compounds are mainly released in the MDF production step and in the end-of-life stage (in the process of making particle board with pine and poplar wood waste). For cardboard boxes, these chemicals are mainly released in the corrugated cardboard manufacturing process. In RPCs, the detergent used in the washing step is the main contributor to human toxicity, followed by the production of granulated polypropylene. Most of the energy embedded in plastic and cardboard boxes comes from non-renewable sources, mainly fossil fuels and nuclear power. In wooden boxes, renewable and non-renewable sources are more balanced, although the proportion of non-renewable energy is larger. The wooden and cardboard boxes required more energy per functional unit than the RPCs, 7.63 MJ and 0.55 MJ, respectively (Table 7 and Table 8). These energies were needed for the extraction of box building materials (wood and pulp) and manufacturing, as compared with the plastic crates that only demanded 0.18 MJ (Table 6). That value is much lower because plastic boxes have 150 rotations during their life cycle whilst for wooden and cardboard boxes it is necessary to produce more materials in the manufacture of new wooden and cardboard boxes, as they are single-use items. The high value of renewable biomass demand for wooden boxes can be explained by the gross calorific energy embedded in the wood used to manufacture wooden boxes.

**Figure 5:**
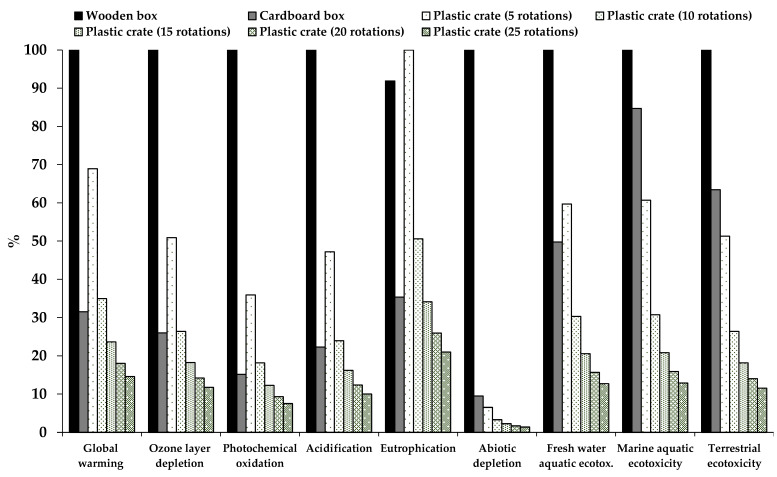
Comparison between single-use wooden and cardboard boxes, and reusable plastic crates with a different number of rotations.

Other studies assessing the environmental impact of different types of boxes reached conclusions similar to those presented in our study. Lo-Iacono-Ferreira et al. [47] performed an LCA of different cardboard boxes used to transport fruit and vegetables to different countries and with different end-of-life scenarios. They calculated the global warming potential of each type of box and concluded that the highest impact was linked to the manufacture of cardboard boxes, followed by the transport. On the other hand, in their study, the impact on climate change of the end-of-life stage was found to be negligible, considering that in this scenario nearly 87% of the cardboard boxes are recycled. In our study, by recycling cardboard boxes the carbon footprint showed a 26% decrease. Del Borghi et al. [48] used the LCA approach to compare the impact of RPCs and wooden and cardboard boxes used for food transport. They concluded that the reuse of plastic crates led to a reduction in greenhouse gas emissions compared with single-use plastic crates, thereby reducing the carbon footprint by 96%. In their study, the life cycle of corrugated cardboard contributed the most to the eutrophication potential in comparison with wooden and plastic crates, mainly because of the wastewater from cardboard production. Our results show a larger eutrophication potential in the case of wooden and cardboard boxes compared with RPCs, mainly due to the box production step. In accordance with our results, Abejón et al. [2] also concluded that RPCs have a significantly lower environmental impact than single-use cardboard boxes. In their study, the stages with the highest impact in the case of the cardboard boxes were the manufacturing stage and the recovery of the paper fibers at the end-of-life, while for RPCs the highest environmental impact was linked to sanitation and transport. In our case, the impact of RPCs was caused by crate production and cleaning, whilst the impact due to crate transport was negligible (the contribution in the assessed impact categories was in the range of 2–8%). The consumption of materials avoided by the recycling processes has a beneficial effect on the environment. The recycling of materials during the waste treatment of cardboard boxes and RPCs reduced the impact in all the impact categories assessed, except for ozone layer depletion and eutrophication potential in RPCs. Tua et al. [49] evaluated the environmental performance of RPCs with a different number of rotations (uses) and concluded that a minimum of three rotations is required to improve sustainability, obtaining a 65% carbon footprint reduction. In our scenario, if we change the number of rotations to three we obtain the same reduction in the carbon footprint (65.6%), but a minimum of approximately 15 rotations would be necessary to reduce all the impacts in comparison to single-use cardboard and wooden boxes (Figure 5). Accorsi et al. [35] compared the economic and environmental impact of single-use wooden and corrugated cardboard boxes to that of RPCs from production until the end-of-life in different scenarios. They obtained that the transport stage affected the sustainability of the reusable plastic crates, while for single-use boxes the principal contributor to the environmental impacts was the manufacturing phase. Similar to our results, in their study, the RPC system led to lower emissions in terms of CO2eq. Albrecht et al. [24] also used the LCA methodology to study the environmental impact of RPCs and single-use wooden and cardboard boxes. Similar to our study, they concluded that the principal contribution to the environmental impacts in single-use boxes (wooden and cardboard) and RPCs is caused by the manufacturing phase. In their study, the activity with the second-greatest impact was the end-of-life in the case of wooden and cardboard, and service life (which involves delivery to the retailer, take-back, inspection, and washing) for RPCs. In our study, the end-of-life was also the second main contributor in the case of wooden boxes, but not for cardboard boxes. The washing step of RPCs also showed a significant impact in our study.

## 4. Conclusions

The results obtained highlight the risk of fresh produce cross-contamination with pathogenic microorganisms via food-handling containers. Cross-contamination of cauliflowers was more widespread when it occurred via polypropylene than via cardboard or MDF. Furthermore, the survival potential of *Salmonella* under supply chain conditions in the contaminated contact materials and the cross-contaminated cauliflower was demonstrated. The LCA performed showed that RPCs are a better choice to reduce the environmental impacts than single-use cardboard and wooden boxes. The RPCs obtained the lowest impact values for all the categories. Operations used to obtain raw materials for manufacturing wooden and corrugated cardboard boxes have a large impact on marine and terrestrial ecotoxicities and acidification categories. Therefore, the use of RPCs is environmentally beneficial; in fact, in our scenario, a service life of only 15 rotations was sufficient to reduce all the impacts in comparison with single-use cardboard and wooden boxes. However, the hygiene of these reusable containers must be properly maintained to reduce food safety risks.

## Figures and Tables

**Table 1:** Sampling plan of the experiment on transfer and survival of *Salmonella* via polypropylene. Types of samples analyzed at different sampling times.

Sample Type	Sampling Time (Days)						
	0	0.1	1	3	6	9	13
IC ^a^ before contact with PP pieces	X						
IC after contact with PP pieces	X	X	X	X	X	X	X
PP pieces after contact with IC	X						
PP pieces after contact with IC and non-IC	X	X	X	X	X	X	X
Non-IC before contact with PP pieces	X						
Cross-contaminated cauliflower ^b^	X	X	X	X	X	X	X
Polyethylene film from IC		X	X	X	X	X	X
Polyethylene film from cross-contaminated cauliflower		X	X	X	X	X	X

PP: Polypropylene. ^a^ Inoculated cauliflower. ^b^ Non-inoculated cauliflower after contact with PP pieces contaminated previously by contact with inoculated cauliflowers. Samples were stored for seven days at 4 °C plus six days at 8 °C.

**Table 2:** Life cycle inventory of plastic, cardboard, and wooden boxes. MDF: medium density fiberboard.

Type of Box	Materials	Weight (kg)
Plastic	Polypropylene	1.550
Cardboard	Corrugated Cardboard	0.440
Wooden	All	1.586
	MDF	1.032
	Pinewood	0.330
	Poplar wood	0.221
	Stainless Steel	0.003

**Table 3:** Transport network for single-use cardboard and wooden boxes and reusable plastic crates (RPCs) from manufacture to the end-of-life scenario.

Type of Box	Phase	Mean Distance (km)	Reference
RPCs	Material–manufacturing plant	1000	[2]
RPCs	Manufacturing–packaging center	500	[2]
Cardboard boxes	Material–manufacturing plant	467	[33]
Cardboard boxes	Manufacturing–packaging center	50	[33]
Wooden boxes	Material–manufacturing plant	400	[34]
Wooden boxes	Manufacturing–packaging center	100	[34]
All	Packaging center–logistics platform	400	[2]
All	Logistics platform–retailer	100	[2]
All	Retailer–logistics platform	100	[2]
RPCs	Logistics platform–washing center	100	[2]
RPCs	Washing center–packaging center	200	[2]
RPCs	Washing center–End-of-life	650	[2]
Cardboard boxes	Logistics platform–End-of-life	100	[2]
Wooden boxes	Logistics platform–End-of-life	100	[35]

**Table 4:** Proportion of positive samples (Number of positive samples/Number of samples analyzed) for each type of sample in the experiment of transfer and survival via polypropylene. Storage for seven days at 4 °C plus six days at 8 °C. NA: Not analyzed.

Sample Type	Sampling Time (Days)							Total (*)
	0	0.1	1	3	6	9	13	
Inoculated cauliflower	3/3	3/3	3/3	3/3	3/3	3/3	3/3	21/21 (100%)
PP pieces	3/3	3/3	3/3	3/3	3/3	2/3	1/3	18/21 (33%)
Cross-contaminated cauliflower	3/3	2/3	3/3	3/3	2/3	2/3	2/3	17/21 (35%)
Polyethylene film from inoculated cauliflower	NA	3/3	3/3	3/3	3/3	3/3	2/3	17/18 (71%)
Polyethylene film from cross-contaminated cauliflower	NA	0/3	1/3	0/3	0/3	0/3	0/3	1/18 (0%)

* % of the positive samples detected by direct plating.

**Table 5:** Prevalence (mean ± standard deviation (proportion of positive samples after enrichment)) of *Salmonella enterica* in container pieces and cross-contaminated cauliflowers during storage (seven days at 4 °C plus six days at 8 °C). Data expressed in log cfu/cm^2^ for the container materials, and in log cfu/g for the cauliflowers. PP: polypropylene.

Storage Time (days)	PP	Cardboard	Fiberboard	Cross-Contaminated Cauliflower (PP)	Cross-Contaminated Cauliflower (Cardboard)	Cross-Contaminated Cauliflower (Fiberboard)
0	0.49 ± 0.71 (3/3)	1.14 ± 0.83 (3/3)	0.10 ± 0.40 (3/3)	<0.7 (3/3)	<0.7 (2/3)	<0.7 (2/3)
1	0.40 ± 0.92 (3/3)	0.71 ± 0.15 (3/3)	<−0.14 (3/3)	0.85 ± 0.21 (3/3)	<0.7 (0/3)	<0.7 (0/3)
6	0.20 ± 0.35 (3/3)	<−0.14 (3/3)	<−0.14 (3/3)	<0.7 (2/3)	<0.7 (0/3)	<0.7 (0/3)
13	<−0.14 (1/3)	<−0.14 (3/3)	<−0.14 (3/3)	0.90 ± 0.17 (2/3)	<0.7 (0/3)	<0.7 (0/3)

**Table 6:** Life cycle impact per functional unit in reusable plastic crates.

Impact Category	Unit	Total	Plastic Crate Production	Cleaning	Transport	End-of-Life *
**Global warming**	kg CO_2_ eq	6.02 × 10^−3^	73%	22%	5%	−25%
**Ozone layer depletion**	kg CFC-11 eq	1.03 × 10^−9^	39%	54%	6%	1%
**Photochemical oxidation**	kg C_2_H_4_ eq	1.30 × 10^−6^	77%	20%	3%	−23%
**Acidification**	kg SO_2_ eq	2.59 × 10^−5^	70%	26%	4%	−16%
**Eutrophication**	kg PO_4_^3−^ eq	1.22 × 10^−5^	64%	26%	3%	7%
**Abiotic depletion**	kg Sb eq	7.82 × 10^−8^	65%	26%	9%	−20%
**Fresh water aquatic ecotoxicity**	kg 1,4-DB eq	3.23 × 10^−3^	67%	31%	2%	−3%
**Marine aquatic ecotoxicity**	kg 1,4-DB eq	7.27 × 10^0^	69%	30%	2%	−2%
**Terrestrial ecotoxicity**	kg 1,4-DB eq	1.55 × 10^−5^	48%	49%	3%	0%
**Human toxicity**	kg 1,4-DB eq	3.46 × 10^−3^	69%	27%	4%	−17%
**Total energy (non-renewable and renewable)**	MJ	1.77 × 10^−1^	70%	28%	2%	−35%
**Total non-renewable**	MJ	1.48 × 10^−1^	75%	22%	2%	−39%
Non-renewable, fossil fuels	MJ	1.09 × 10^−1^	84%	13%	3%	−45%
Non-renewable, nuclear	MJ	3.92 × 10^−2^	35%	64%	0%	−8%
Non-renewable, biomass	MJ	3.68 × 10^−6^	64%	32%	4%	4%
**Total renewable**	MJ	1.80 × 10^−2^	39%	61%	0%	−3%
Renewable, biomass	MJ	4.89 × 10^−3^	61%	39%	1%	−7%
Renewable, wind, solar, geothermal	MJ	6.13 × 10^−3^	20%	80%	0%	−1%
Renewable, water	MJ	7.00 × 10^−3^	40%	59%	1%	0%

* The negative value in end-of-life is due to material avoided in the recycling process.

**Table 7:** Life cycle impact per functional unit in single-use cardboard boxes.

Impact Category	Unit	Total	Cardboard Box Production	Transport	End-of-Life *
**Global warming**	kg CO_2_ eq	5.88 × 10^−2^	92%	8%	−26%
**Ozone layer depletion**	kg CFC−11 eq	7.54 × 10^−9^	88%	12%	−24%
**Photochemical oxidation**	kg C_2_H_4_ eq	1.22 × 10^−5^	95%	5%	−36%
**Acidification**	kg SO_2_ eq	2.59 × 10^−4^	92%	8%	−23%
**Eutrophication**	kg PO_4_^3−^ eq	9.62 × 10^−5^	97%	3%	−50%
**Abiotic depletion**	kg Sb eq	2.35 × 10^−6^	97%	3%	−48%
**Fresh water aquatic ecotoxicity**	kg 1,4-DB eq	5.56 × 10^−2^	99%	1%	−49%
**Marine aquatic ecotoxicity**	kg 1,4-DB eq	2.15 × 10^2^	99%	1%	−47%
**Terrestrial ecotoxicity**	kg 1,4-DB eq	3.04 × 10^−4^	98%	2%	−44%
**Human toxicity**	kg 1,4-DB eq	8.29 × 10^−1^	97%	3%	−47%
**Total energy (non-renewable and renewable)**	MJ	5.53 × 10^−1^	94%	6%	−71%
**Total non-renewable**	MJ	9.32 × 10^−1^	92%	8%	−26%
Non-renewable, fossil fuels	MJ	8.29 × 10^−1^	91%	9%	−26%
Non-renewable, nuclear	MJ	1.02 × 10^−1^	99%	1%	−26%
Non-renewable, biomass	MJ	2.40 × 10^−4^	100%	0%	−62%
**Total renewable**	MJ	1.38 × 10^−1^	100%	0%	−79%
Renewable, biomass	MJ	1.08 × 10^−1^	100%	0%	−82%
Renewable, wind, solar, geothermal	MJ	7.73 × 10^−3^	99%	1%	−45%
Renewable, water	MJ	2.15 × 10^−2^	98%	2%	−33%

* The negative value in end-of-life is due to material avoided in the recycling process.

**Table 8:** Life cycle impact per functional unit in single-use wooden boxes.

Impact Category	Unit	Total	Wooden Box Production				Transport	End-of-Life
			MDF	Pine	Poplar	Stainless Steel		
**Global warming**	kg CO_2_ eq	1.86 × 10^−1^	51%	9%	3%	1%	14%	23%
**Ozone layer depletion**	kg CFC−11 eq	2.90 × 10^−8^	42%	7%	3%	1%	16%	32%
**Photochemical oxidation**	kg C_2_H_4_ eq	8.07 × 10^−5^	55%	6%	5%	1%	4%	28%
**Acidification**	kg SO_2_ eq	1.16 × 10^−3^	42%	7%	2%	1%	9%	40%
**Eutrophication**	kg PO_4_^3−^ eq	2.50 × 10^−4^	70%	11%	4%	2%	10%	5%
**Abiotic depletion**	kg Sb eq	2.47 × 10^−5^	89%	2%	1%	0%	2%	5%
**Fresh water aquatic ecotoxicity**	kg 1,4-DB eq	1.12 × 10^−1^	57%	5%	1%	3%	4%	30%
**Marine aquatic ecotoxicity**	kg 1,4-DB eq	2.53 × 10^2^	45%	5%	1%	5%	3%	40%
**Terrestrial ecotoxicity**	kg 1,4-DB eq	4.80 × 10^−4^	67%	11%	7%	2%	8%	6%
**Human toxicity**	kg 1,4-DB eq	2.47 × 10^−1^	41%	4%	1%	1%	5%	48%
**Total energy (non-renewable and renewable)**	MJ	7.63 × 10^0^	41%	28%	17%	0%	5%	8%
**Total non-renewable**	MJ	4.54 × 10^0^	39%	5%	2%	1%	9%	45%
Non-renewable, fossil fuels	MJ	4.05 × 10^0^	40%	5%	2%	0%	10%	43%
Non-renewable, nuclear	MJ	4.86 × 10^−1^	30%	3%	3%	1%	1%	61%
Non-renewable, biomass	MJ	1.35 × 10^−4^	49%	22%	26%	0%	2%	−65%
**Total renewable**	MJ	3.09 × 10^0^	31%	43%	26%	0%	0%	−31%
Renewable, biomass	MJ	2.96 × 10^0^	30%	43%	26%	0%	0%	−33%
Renewable, wind, solar, geothermal	MJ	3.79 × 10^−2^	53%	5%	2%	1%	2%	38%
Renewable, water	MJ	9.87 × 10^−2^	38%	8%	10%	1%	3%	41%

## Data Availability

The data presented in this study are available upon request from the corresponding author.

## Supplementary Materials

The following are available online at https://www.mdpi.com/article/10.3390/foods10061254/s1. Table S1: Characteristics of the cauliflowers used in the experiments, Table S2: Detailed assessment of the transfer of *Salmonella* from pieces of different materials to non-inoculated cauliflowers.
