# Supplementary material for: Reusable Plastic Crates (RPCs) for Fresh Produce (Case Study on Cauliflowers): Sustainable Packaging but Potential Salmonella Survival and Risk of Cross-Contamination
Source: Foods. 2021 Jun 1;10(6):1254. doi: 10.3390/foods10061254 (PMC8228333; doi:10.3390/foods10061254)
Supplement: Supplementary file 1 [file foods-10-01254-s001.zip › foods-1232826-supplementary.pdf]

Supplementary information

Table S1. Characteristics of the cauliflowers used in the experiments. SD: standard deviation. *Measurement from stem cut to apex.

Parameter	Mean ± SD	Units
Weight	308 ± 72	g
Height*	7.7 ± 0.9	cm
Diameter	9.4 ± 0.7	cm

Table S2. Detailed assessment of the transfer of *Salmonella* from pieces of different materials to non-inoculated cauliflowers.

Experiment	cfu/piece of box or crate	Predicted cfu/g in cross-contaminated cauliflowers	Observed cfu/g in cross-contaminated cauliflowers
PP	38	0.25	<5
Cardboard	169	1.1	<5
Fiberboard	15	0.1	<5

Assumptions and data for the calculations: All the cfu are transferred from the pieces to the non-inoculated cauliflowers. There is a homogeneous distribution of the cfu in the non-inoculated cauliflowers. Weight of the apical half of the cauliflowers: 300 g/2= 150 g. Surface area of the pieces = 12.25 cm^2. PP: polypropylene.

Experiment with PP

The mean number of cfu in a PP piece was $10^{0.49}$ cfu/cm^2 * 12.25 cm^2≈38 cfu. Assuming that all the cfu are transferred from the PP piece to the non-inoculated cauliflowers, a homogeneous distribution in the non-inoculated cauliflowers, and estimating a weight of 150 g for the apical half of the cauliflowers (see S1) the population in the cross-contaminated cauliflowers would be 38 cfu/150 g≈ 0.25 cfu/g. In this case, the result of the analysis indicated a level of *Salmonella* <5 cfu/g in the cross-contaminated cauliflowers, which concurs with the prediction based on the level detected in the PP pieces.

Experiment with cardboard

The mean number of cfu in a cardboard piece was $10^{1.14}$ cfu/cm^2 * 12.25 cm^2≈169 cfu. Assuming that all the cfu are transferred from the PP piece to the non-inoculated cauliflowers, a homogeneous distribution in the non-inoculated cauliflowers, and estimating a weight of 150 g for the apical half of the cauliflowers (see S1) the population in the cross-contaminated cauliflowers would be 169 cfu/150 g≈ 1.1 cfu/g. In this case, the result of the analysis indicated a level of *Salmonella* <5 cfu/g in the cross-contaminated cauliflowers, which concurs with the prediction based on the level detected in the cardboard pieces.

Experiment with fiberboard

The mean number of cfu in a fiberboard piece was $10^{0.1}$ cfu/cm^2 * 12.25 cm^2≈15 cfu. Assuming that all the cfu are transferred from the PP piece to the non-inoculated cauliflowers, a homogeneous distribution in the non-inoculated cauliflowers, and estimating a weight of 150 g for the apical half of the cauliflowers (see S1) the population in the cross-contaminated cauliflowers would be 15 cfu/150 g≈ 0.1 cfu/g. In this case, the result of the analysis indicated a level of *Salmonella* <5 cfu/g in the cross-contaminated cauliflowers, which concurs with the prediction based on the level detected in the fiberboard pieces.
